# Gefitinib, an EGFR Tyrosine Kinase inhibitor, Prevents Smoke-Mediated Ciliated Airway Epithelial Cell Loss and Promotes Their Recovery

**DOI:** 10.1371/journal.pone.0160216

**Published:** 2016-08-17

**Authors:** Monica Valencia-Gattas, Gregory E. Conner, Nevis L. Fregien

**Affiliations:** Department of Cell Biology, University of Miami Miller School of Medicine, Miami, Florida, United States of America; University of Alabama at Birmingham, UNITED STATES

## Abstract

Cigarette smoke exposure is a major health hazard. Ciliated cells in the epithelium of the airway play a critical role in protection against the noxious effects of inhaled cigarette smoke. Ciliated cell numbers are reduced in smokers which weakens host defense and leads to disease. The mechanisms for the loss of ciliated cells are not well understood. The effects of whole cigarette smoke exposure on human airway ciliated ciliated cells were examined using *in vitr*o cultures of normal human bronchial epithelial cells and a Vitrocell^®^ VC 10^®^ Smoking Robot. These experiments showed that whole cigarette smoke causes the loss of differentiated ciliated cells and inhibits differentiation of ciliated cells from undifferentiated basal cells. Furthermore, treatment with the epidermal growth factor receptor (EGFR) tyrosine kinase inhibitor, Gefitinib, during smoke exposure prevents ciliated cell loss and promotes ciliated cell differentiation from basal cells. Finally, restoration of ciliated cells was inhibited after smoke exposure was ceased but was enhanced by Gefitinib treatment. These data suggest that inhibition of EGFR activity may provide therapeutic benefit for treating smoke related diseases.

## Introduction

Cigarette smoke exposure is a major cause of death and disease. In the United States, more than 480,000 deaths per year are attributable to cigarette smoking. In addition, cigarette smoking is a significant risk factor in a variety of lung diseases including chronic obstructive pulmonary disease (COPD), as well as cardiovascular disease and many cancers. Despite the strong relationships of cigarette smoke and these diseases, the mechanisms by which cigarette smoke causes them are not completely understood [1].

The epithelial cells that line the airway provide the primary barrier between the internal tissues of the body and the inhaled tobacco smoke. As a result of their location, these cells are exposed to the highest smoke levels and are therefore at the greatest risk for cellular damage from smoke. In addition to the physical barrier, the ciliated cells and secretory goblet cells in the epithelium protect the airway against the toxic affects of the cigarette smoke by process of mucociliary clearance (MCC) [2, 3]. Goblet cells secrete mucus that floats on the surface of the airway and traps the noxious agents in the inhaled smoke. The beating cilia on the ciliated cells then expel the tainted mucus out of the airway to minimize damage. Efficient MCC requires a balance between the amount of mucus secreted and the numbers of ciliated cells. Overproduction of mucus and/or insufficient numbers of ciliated cells to propel the mucus out of the airway lead to inefficient clearance, accumulation of contaminated mucus and prolonged exposure of the epithelial cells to the harmful inhaled toxins and further hampering MCC and ultimately resulting in disease. Therefore, understanding the basis for these changes in the epithelial cells may help in the development of therapies that can prevent them as well as restore the epithelium in already compromised individuals to prevent further disease.

The correlation between cigarette smoking and reduced numbers of ciliated cells has been known for many years [4], however the mechanism for this has not been entirely worked out. Many previous studies have shown that cigarette smoke extract (CSE) and cigarette smoke condensate (CSC) induce changes of mucin gene expression in airway epithelial cell lines by activating epidermal growth factor receptor (EGFR) signaling [5–7]. CSE has also been shown to impair ciliogenesis [8], to suppress ciliary protein gene expression and cilia length [9, 10] and that EGF downregulates genes needed for ciliogenesis [11]. In this study, we treated primary human bronchial epithelial cells during and after ciliated cell differentiation with whole cigarette smoke (WCS) using a smoke robot system, that more accurately simulates *in vivo* smoke exposure, with the goal of determining whether direct WCS exposure affects differentiation and maintenance of ciliated epithelial cells *in vitro* similar to CSE [8, 12], and if so, whether it is mediated by EGFR activation.

## Materials and Methods

### Chemicals

The EGFR inhibitor Gefitinib (CAS 184475-35-2) was purchased from Cayman Chemicals (Ann Arbor, MI). All other chemicals were purchased from Sigma-Aldrich (St. Louis, MO) unless otherwise stated.

### Cell Culture and Smoke Exposure

Normal human bronchial epithelial (NHBE) cells were obtained from organ donors whose lungs were rejected for transplant. Consent was obtained through the Life Alliance Organ Recovery Agency of the University of Miami, according to protocols approved by the Institutional Review Board. Epithelial cells from the lower trachea and upper bronchi were isolated and cultured as previously described [13–16]. Differentiated cultures were maintained at the air liquid interface cultures for at least 3 weeks before starting experiments.

### Cigarette Smoke Exposure

NHBE cells were exposed to whole cigarette smoke using the Vitrocell^®^ VC 10^®^ Smoking Robot. This system mimics real life CS exposure. Triplicate cultures were treated with smoke from 3R4F research grade cigarettes (University of Kentucky, Lexington, Kentucky USA) using a VITROCELL^®^ VC 10® Smoking Robot with a 35 ml puff volume, 2 s duration and 1 min between puffs or air as a control. For differentiated cells, smoking was done every 2 d for 5 d (3 exposures) and samples were collected 48 h after smoking. During differentiation, NHBE cells were exposed to smoke from 1 cigarette 3 times per week and samples were collected after 14, 21 and 27 days. The cigarette smoke doses used were doses that did not alter cell viability (supplementary data).

### Quantitative RT-PCR (qRT-PCR)

Total RNA was isolated using EZNA RNA isolation kit (Omega Biotek, Norcross, GA) and cDNA was synthesized using the iScript cDNA Synthesis Kit (BioRad, Hercules, CA). Quantitative PCR amplification was performed using the BioRad CFX 96 Real Time System (BioRad, Hercules, CA) and TaqMan Universal Assays (Applied Biosystems, Branchburg, NJ) including gene-specific primers and probe sets designed for Foxj1 (HS00230964_m1), Multicilin (MCIN, HS04234534_m1), GAPDH (Hs99999905_m1) and β-2 microglobulin (B2M, Hs99999907_m1). Relative mRNA amounts were calculated by normalizing the targeted molecules to an internal control (GAPDH or B2M) ΔCt method.

### Neutral Red Viability Assay

Differentiated NHBE were exposed to either WCS from 3RF4 cigarettes or air (control) from the indicated number of cigarettes every two days, before feeding, and assayed for viability 24 h later. The cells were washed once with pre-warmed PBS and 40 μg/ml neutral red reagent in ALI media was added to the basolateral side and incubated at 37°C with 5% CO_2_ for 4 hours. Cells were then washed both apically and basolaterally with PBS and the neutral red dye was extracted from the viable cells using an acidified ethanol solution (50% ethanol, 49% deionized water, 1% glacial acetic acid). The amount of the solubilized dye was quantified by measuring the optical absorbance at 540nm (A_540_) using a spectrophotometer. Percent viability was calculated by dividing the A_540_ from smoke treated cells by the A_540_ from control air treated cells.

### Immunofluorescence

NHBE cells on Transwell^®^ filters were fixed in 4% paraformaldehyde in PBS, pH 7.4 for 15 min and permeabilized with 1% Triton X-100 in PBS for 20 min at room temperature. After permeabilization cells were washed with PBS and blocked with 3% BSA in PBS for 1 hour at room temperature followed by goat anti-human FoxJ1 antibody (R&D Systems Minneapolis, MN, 0.2 mg/ml; diluted 1:200) and mouse anti-human acetylated α-tubulin (Sigma, St. Louis, MO, diluted 1:2000) in blocking solution and incubated overnight at 4°C. Nuclei were labeled with 4,6-diamidine-2-phenylindole (DAPI, KPL, Gaithersburg, MD). Samples on Transwell^®^ membranes were mounted on slides with Fluoro-Gel (EMS, Hatfield, PA) and fluorescent images were acquired on a Leica DM6000 microscope with a SP5 confocal module at the University of Miami McKnight Analytical Imaging Core Facility. Extended focus 2D images were generated using Volocity Software version 6.1.1 software (Perkin-Elmer, Waltham, MA). Total nuclei and FoxJ1 positive nuclei in confocal images were counted using the find objects feature in Volocity Quantitation software (PerkinElmer, Waltham, MA) taking into account touching objects and objects by size. The percent FoxJ1 positive (FoxJ1+) cells was calculated by dividing FoxJ1+ nuclei by total nuclei x 100. Measurements were taken from 3–4 random locations of each Transwell membranes and using triplicate membranes for each condition and cells from 3–7 different lung donors.

### Phospho-EGFR and Phospho-Erk1/2 Western Blotting

Differentiated cells were treated with air (control) or WCS from 2 cigarettes with or without Gefitinib (500 nM) in media. Twenty-four hours later, cells were solubilized using 1% SDS, 10 mM Tris pH 8.3, 0.1 mM EDTA supplemented with cOmplete protease inhibitor cocktail and PhoSTOP phosphatase inhibitor cocktail (Roche, Indianapolis, IN). Following sonication to reduce viscosity and centrifugation (12,000 rpm, 15 min at 4°C), proteins were quantified by BCA assay (Thermo-Fisher, Waltham, MA). Equal amounts of protein (25 μg) from each sample was diluted in buffer (100 mM Tris, pH 6.8, 4% SDS, 20% glycerol, and 100 mM dithiothreitol), separated on a 4–15% Ready Gel Precast SDS–polyacrylamide gels (Bio-Rad, Hercules, CA), and transferred by electrophoresis to PVDF 0.45 μm membranes Amersham Hybond (GE Lifesciences, Marlborough, MA). Membranes were then blocked (5% BSA in 137 mM NaCl, 20 mM Tris pH 7.6, 0.1% Tween-20) rinsed, and incubated with anti-phosphoEGFR rabbit mAb or anti-phospho ERK1/2 rabbit mAb (Cell Signaling, Danvers, MA) and anti-β-Actin monoclonal antibody (clone AC-74, Sigma, St Louis, MO). The membranes were then incubated with HRP-coupled goat anti rabbit IgG (KPL, Gaithersburg, MD). HRP was detected using SuperSignal West Pico Chemiluminescent Substrate (Thermo-Fisher, Waltham, MA), images were acquired using Biorad ChemiDocXRS and intensity signals were quantified and analyzed with ImageLab software (Bio-Rad, Hercules, CA). Blots were stripped and probed with anti-β-Actin mouse monoclonal antibody clone AC-74 (Sigma, St Louis, MO) as a loading control.

### Statistical Analysis

Statistical analysis was done using Prizm 5 software (Graphpad Software, Inc.). Two-tailed student’s T test was used when comparing 2 samples and one-way ANOVA when comparing multiple samples.

## Results

### Whole cigarette smoke exposure causes a reduction of ciliated cells in differentiated NHBE cells

**Fig 1:**
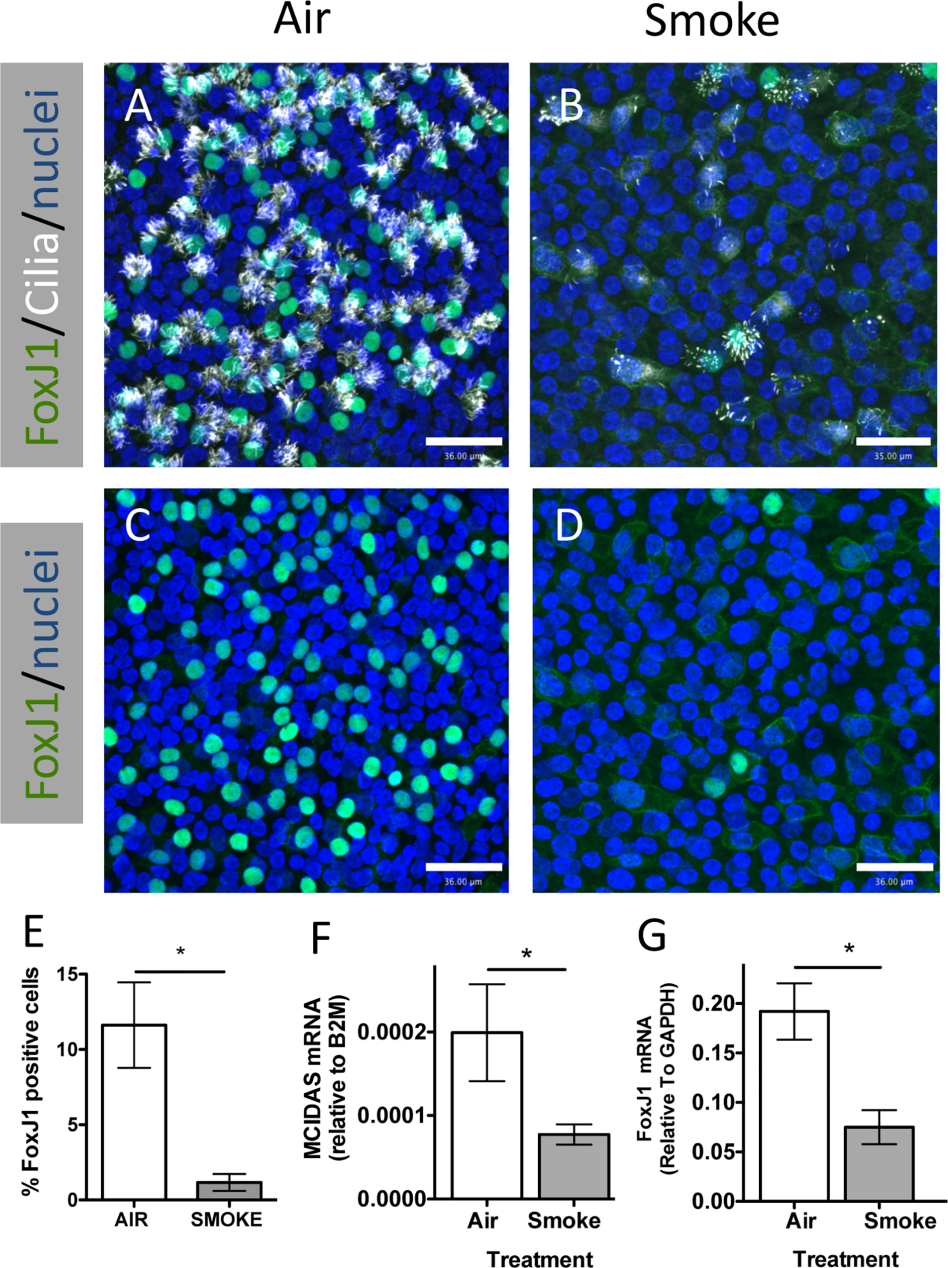
Whole cigarette smoke exposure causes an acute reduction of FoxJ1 positive cells. **(A-D)** Representative confocal micrographs of differentiated human airway epithelial cells treated with Air (A, C) or Smoke (B, D) stained for FoxJ1 (green), acetylated-tubulin (white) or nuclei (blue) (A, B). C and D represent the same microscopic fields as A and B respectively showing only FoxJ1 and nuclear staining. Scale bar = 36 μm **(E)** Quantification of FoxJ1 positive (i.e. ciliated cells) in Air and Smoke treated samples showing the mean and SEM from 3 independent lung donors. * P<0.05, two-tailed Student’s t test. **(F, G)** Duplex qRT-PCR MCIDAS mRNA relative to B2M mRNA **(F)** and FoxJ1 mRNA relative to GAPDH mRNA **(G)** in cells treated with Air (white bars) and Smoke (grey bars). The bars show the mean and SEM of RNA from 3 independent lung donors. P values calculated using two-tailed Student’s t test are shown above the columns.

To determine the effect of WCS treatment on differentiated ciliated cells, NHBE cells from 6 different lung donors were differentiated in ALI culture for 21 d and then exposed to WCS from two 3RF4 research cigarettes 3 times on alternate days and compared to clean air control exposure. This treatment did not significantly affect cell viability (S1 Fig). Two days after the final WCS exposure the cells were fixed and immunofluorescently stained for FoxJ1, a ciliated cell specific transcription factor, and acetylated-tubulin, a marker for cilia. mRNA was isolated from parallel samples. Confocal microscopic analysis of the immunofluorescence staining (Fig 1A–1D) indicated that WCS exposure caused a reduction of FoxJ1 positive nuclei and ciliated cells. In addition, the remaining ciliated cells in the WCS smoke treated samples appeared to have fewer cilia and reduced nuclear FoxJ1 staining. Quantification of FoxJ1 positive nuclei confirmed WCS treated cells had significantly fewer FoxJ1 positive nuclei (Fig 1E) than air treated controls. In addition, the mRNA levels of two ciliated cell specific genes, FoxJ1 and MCIDAS (aka Multicilin) were significantly reduced in WCS treated cells (Fig 1F and 1G). These data suggested that exposure to WCS three times over a seven day period caused an acute reduction in the number of ciliated cells by reducing the expression of MCIDAS and FoxJ1, two genes that are required for ciliogenesis [17, 18].

### Inhibition of epidermal growth factor receptor signaling during WCS exposure prevents ciliated cell loss in differentiated NHBE cells

**Fig 2:**
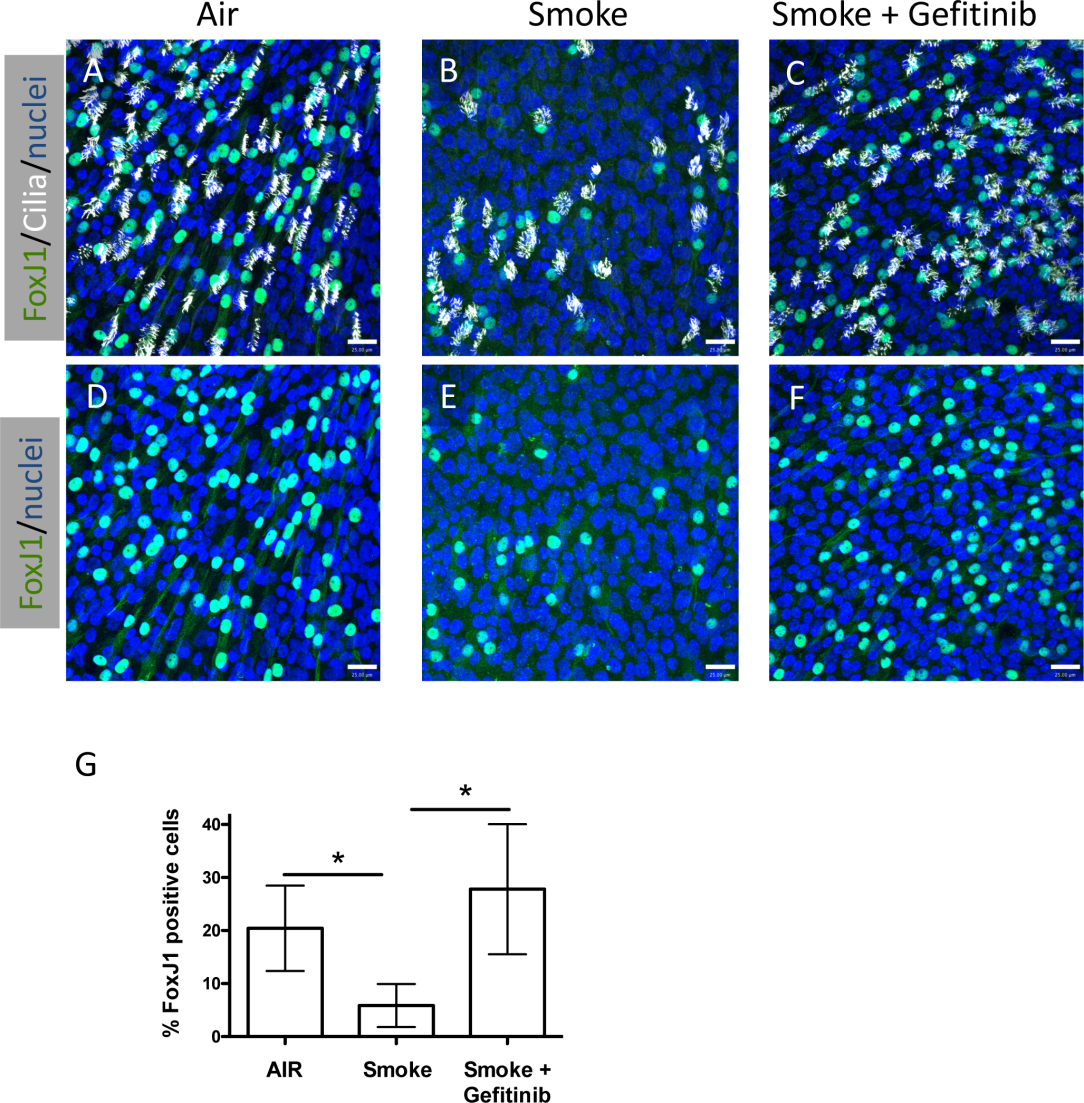
Inhibition of EGFR signaling prevents WCS induced loss of ciliated cells. **(A–F)**, Representative confocal micrographs of differentiated human airway epithelial cells treated with Air **(A, D)**, Smoke **(B, E)** or Smoke + 500 nM Gefitinib **(C, F)** stained for FoxJ1 (green), acetylated-tubulin (white) or nuclei (blue) **(A, B, C)**. **D**, **E** and **F** represent the same microscopic fields as **A**, **B** and **C**, respectively showing only the FoxJ1 and nuclear staining. Scale bar = 25 μm **G**, Quantification of the percent FoxJ1 positive cells relative to the Air treated samples. N = 7 air and smoke, N = 5 smoke + Gefitinib; * P<0.05, one way ANOVA.

Cigarette smoke exposure has been shown to activate EGFR signaling and affect non-ciliated airway epithelial cell gene expression and function [5, 19–21], however the affect of EGFR signaling on ciliated cells has not been reported. To determine whether the reduction of ciliated cells observed after WCS exposures was mediated by EGFR signaling, we inhibited EGFR signaling with a selective inhibitor of the EGFR tyrosine kinase domain, Gefitinib [22] and measured the effect on ciliated cells. Differentiated NHBE cells in ALI culture for 21 days were treated with WCS as described above, with and without Gefitinib (500 nM) in the culture media and compared to NHBE cells that were not treated with WCS (Air control). Gefitinib was added 1 h before WCS exposure was initiated and maintained during the remainder of the experiment. As shown in Fig 2, Gefitinib treatment prevented the WCS-induced loss of FoxJ1 positive nuclei and ciliated cells when compared with non-WCS treated cultures. Preliminary studies showed that addition of Gefitinib had no effect on ciliated cells in air treated controls (S3 Fig). These results suggested that WCS induction of ciliated cell loss may be due to activation of EGFR signaling.

### Whole cigarette smoke exposure induces phosphorylation of EGFR and Extracellular Signal-Regulated Kinase (Erk) 1/2 that is inhibited by Gefitinib

**Fig 3:**
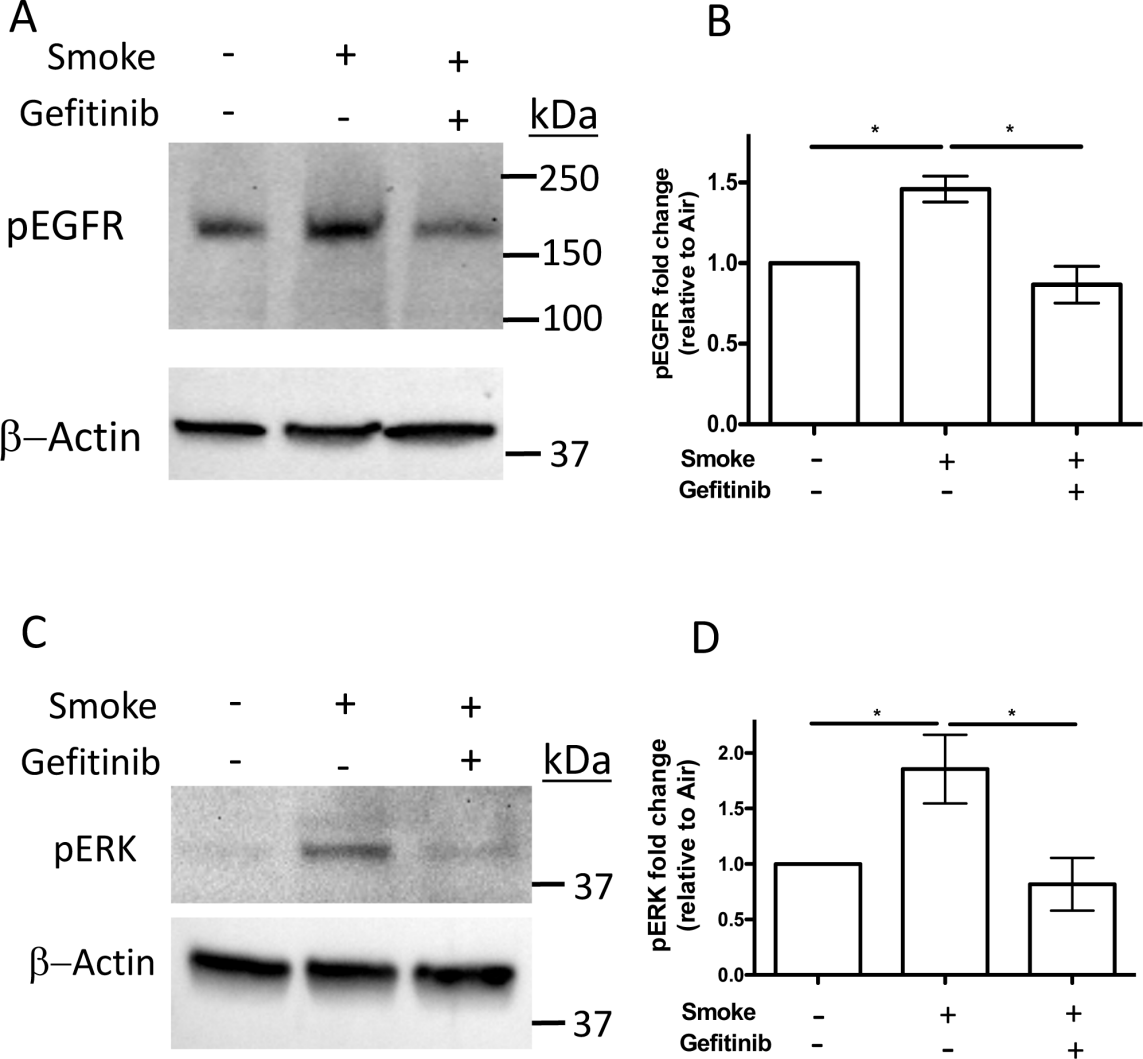
Whole cigarette smoke exposure induces phosphorylation of both EGFR and Erk1/2 that is inhibited by Gefitinib. Differentiated NHBE cells were treated with air or WCS from 2 cigarettes with and without Gefitinib. After 24 h total cell protein lysates were prepared and equal amounts of protein were separated by SDS-PAGE, transferred to PVDF membranes and probed with anti-phospho-EGFR (Tyr1068) (A) or anti-Phospho-Erk1/2 (Thr202/Tyr204) (C) antibodies and anti-β actin antibody as a loading control (A, C). Graphs of the quantification of the signals for phospho-EGFR (B) from 3 different lungs and for phospho-Erk1/2 from 5 different lungs (D) are shown. * P<0.05, one-way ANOVA.

To determine the effect of Gefitinib on EGFR tyrosine kinase activity during WCS exposure, differentiated NHBE cells were treated with WCS from 2 cigarettes with and without Gefitinib or air as a control and the levels of EGFR and Erk1/2 phosphorylation were measured 24 h after exposure by western blotting using anti-phospho-EGFR (Tyr1068) and anti-phospho-Erk1/2 (Thr202/Tyr204) specific antibodies. The results showed that phospho-EGFR (Fig 3A and 3B) and phospho-Erk1/2 (Fig 3C and 3D) were increased in WCS treated cells in the absence of Gefitinib compared to air treated control cells. However, cells treated with WCS in the presence of Gefitinib showed no increase in phospho-EGFR or phospho-Erk1/2 levels. These data suggest that WCS treatment increased EGFR and Erk1/2 phosphorylation by stimulating EGFR tyrosine kinase activity.

### Exposure to whole cigarette smoke inhibits ciliated cell differentiation

**Fig 4:**
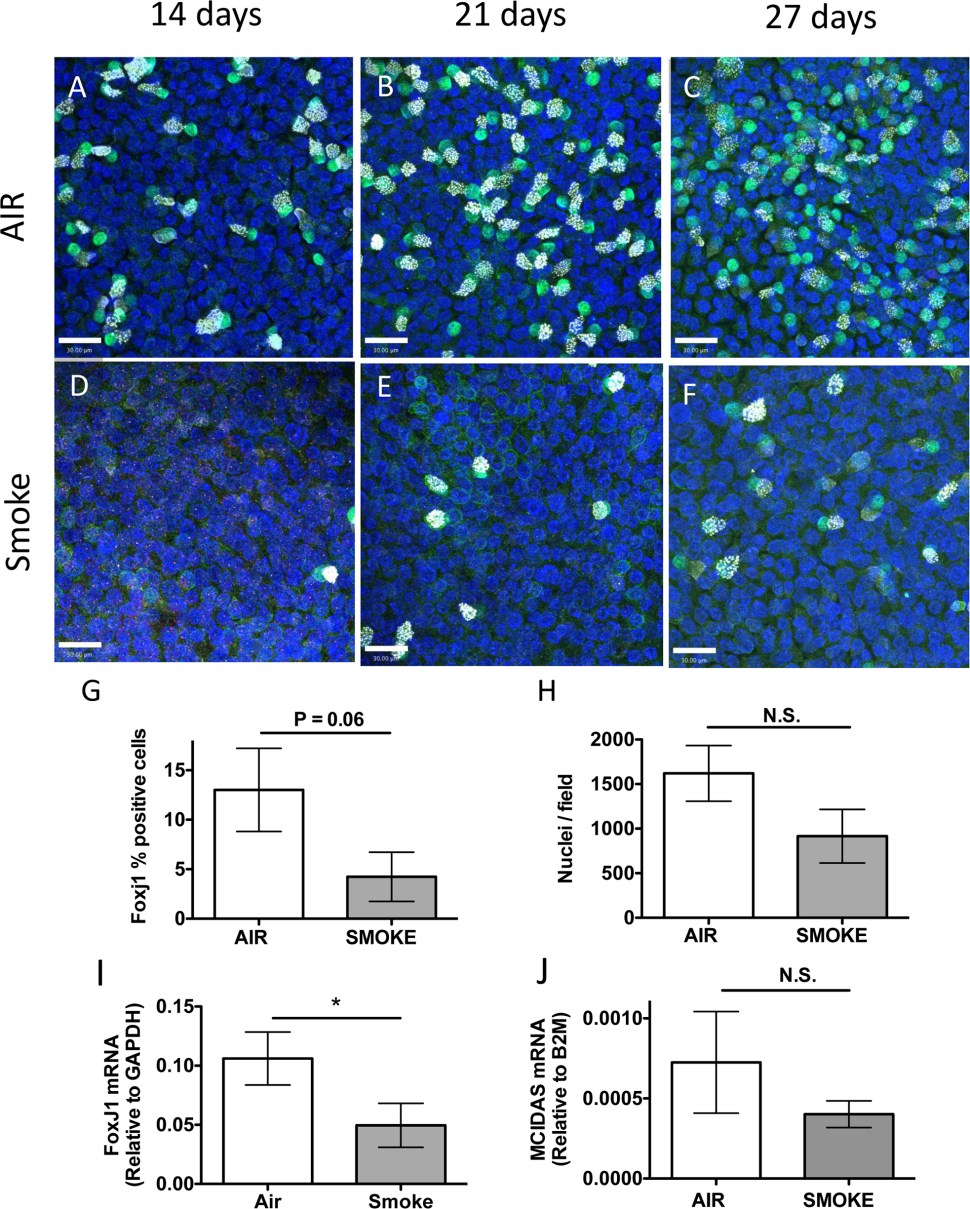
Whole cigarette smoke exposure inhibits ciliated cell differentiation. **(A–F)** Representative confocal micrographs of human airway epithelial cells treated with Air **(A, B, C)** or Smoke **(D, E, F)** for 14 d **(A, D)**, 21 d **(B, E)** or 27 d **(C, F)** during differentiation using ALI conditions and stained for FoxJ1 (green), acetylated-tubulin (white) or nuclei (blue). Scale bar = 30 μm. **(G)** Quantification of FoxJ1 positive cells treated with Air (white bars) or Smoke (grey bars) for 27 d during differentiation in ALI conditions. N = 3 different lung donors, * P = 0.06, two-tailed Student’s t test. **(H)** Quantitation of the average number of nuclei / microscopic field in cells treated with Air (white bar) or WCS (grey bar) for 27 days during differentiation. N = 3 different lung donors, N.S., not significant, two-tailed Student’s t-test. **(I)** Duplex qRT-PCR of FoxJ1 mRNA relative to GAPDH mRNA in cells treated with Air or Smoke for 27 d during differentiation using ALI conditions. N = 8 different lung donors, * P<0.05, two-tailed Student’s t-test. **(J)** Duplex qRT-PCR of MCIDAS mRNA relative to B2M in human airway epithelial cells treated with Air (white bars) or Smoke (grey bars) for 27 d of differentiation in ALI conditions. N = 8 different lung donors, N.S., not significant, two-tailed Student’s t-test.

To test the effect of WCS exposure on ciliated cell differentiation, undifferentiated NHBE cells on Transwell membranes were treated with air or smoke from one, 3RF4 research cigarette, 3 times per week for 4 weeks beginning the day the cells were taken to ALI conditions to initiate differentiation. This exposure paradigm was used because preliminary experiments showed that undifferentiated cells did not survive exposure to two cigarettes, however one cigarette did not affect viability (S2 Fig) suggesting that undifferentiated cells are more sensitive to WCS toxicity than differentiated cells. Cells were fixed after 14, 21 and 27 d of differentiation and immunofluorescently stained for FoxJ1 and acetylated-tubulin to identify ciliated cells. The confocal micrographs (Fig 4A–4F) indicated that FoxJ1 positive nuclei and ciliogenesis were reduced in WCS treated cells compared to air treated control cells. Quantifying the FoxJ1 positive (FoxJ1+) cells in the 27 d cultures confirmed there was a significantly lower percentage of FoxJ1 positive nuclei in the WCS treated cells (4.3 +/- 4.2% vs. 13.0 +/- 7.3%) (Fig 4G), consistent with the immunofluorescence data. Counting of numbers of nuclei per microscopic field suggested there were ~40% fewer cells in the smoke treated samples, however this was not statistically significant (p = 0.2), suggesting that cell growth may have been affected by the WCS treatment (Fig 4H) but this does not account for the reduction in the percentage of FoxJ1 positive cells. In addition, cells treated with WCS for 27 d had significantly less FoxJ1 mRNA (Fig 3I) and a reduction in MCIDAS mRNA (Fig 4J) however, unlike WCS treated differentiated cells the change in MCIDAS mRNA was not statistically significant. These data supported the hypothesis that WCS exposure inhibited differentiation of basal cells into ciliated cells in ALI conditions by reducing FoxJ1 mRNA levels but not MCIDAS mRNA.

### Inhibition of EGFR signaling allows ciliated cell differentiation during WCS exposure

**Fig 5:**
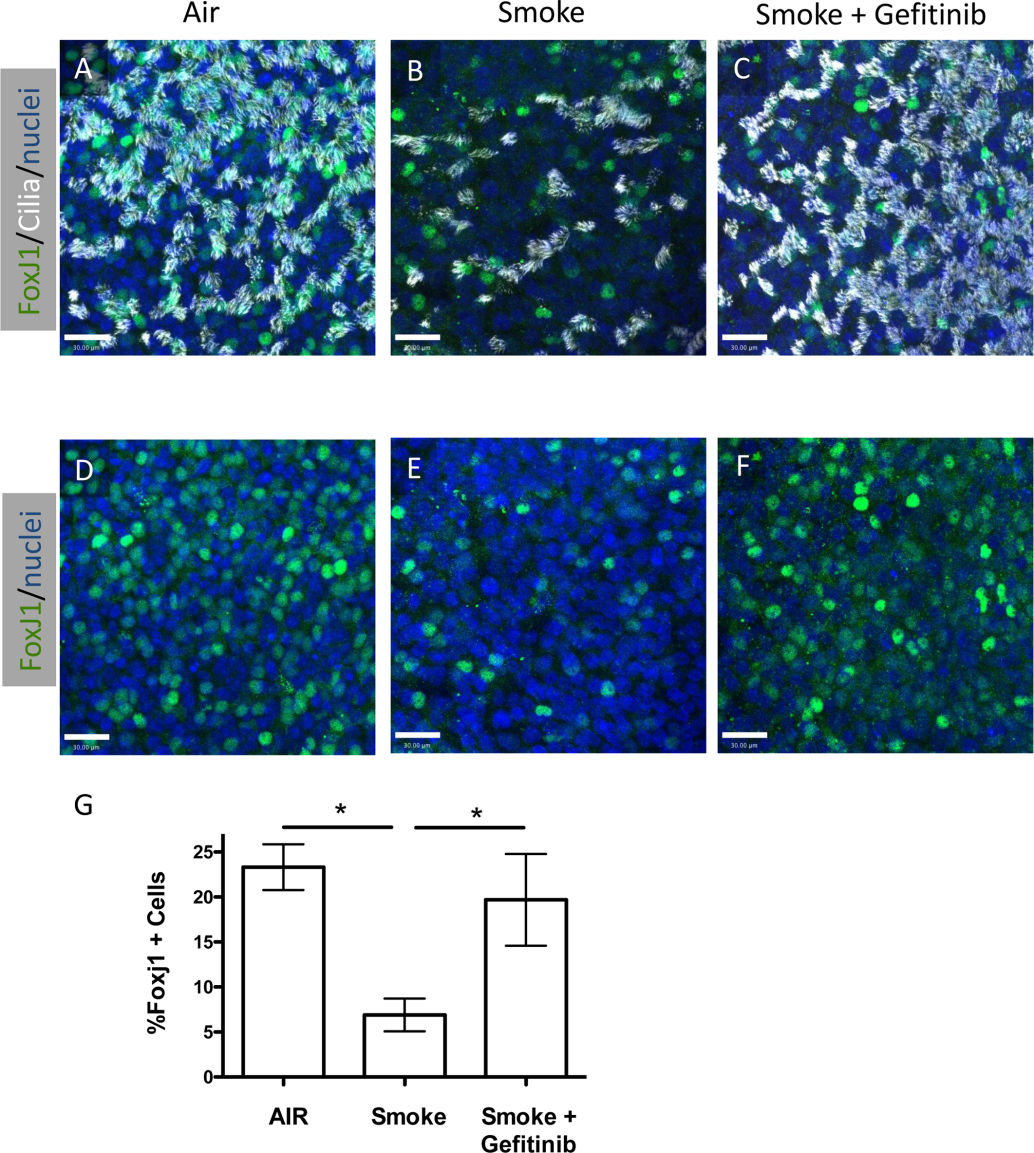
Inhibition of EGFR signaling restores ciliated cell differentiation during whole cigarette smoke exposure. **(A–F)** Representative confocal micrographs of human airway epithelial cells after treatment with Air **(A, D)**, Smoke **(B, E)** or Smoke + Geftinib **(C, F)** during 21 d of differentiation using ALI conditions, stained for FoxJ1 (green), acetylated-tubulin (white) or nuclei (blue) **(A, B, C)**. Panels **D**, **E** and **F** represent the same microscopic fields as **A**, **B** and **C**, respectively showing only the FoxJ1 and nuclear staining. Scale bar = 30 μm **(G)** Quantification of the percent FoxJ1 positive cells after 21 d of differentiation using ALI conditions treated with Air, Smoke or Smoke + Gefitinib. N = 7 different lung donors, * P<0.05, one-way ANOVA.

Since the EGFR inhibitor Gefitinib prevented WCS-induced reduction of differentiated ciliated cells, we next tested whether Gefitinib could restore ciliated cell differentiation blocked during WCS exposure. In our initial attempts to do this experiment, we initiated WCS exposure with or without Gefitinib on the day cells were switched to ALI conditions as done in the previous experiments. However, the addition of Gefitinib caused a rapid loss of undifferentiated cells even in the absence of WCS exposure suggesting that EGFR activity is required to support undifferentiated cell survival in the early stages of differentiation. We then tested whether the cells could survive if Gefitinib was added later after initiating ALI conditions and found that Gefitinib could be added 7 days after initiating ALI without causing cell loss (data not shown). The protocol was changed to start WCS exposure using one cigarette every 2 d with or without Gefitinib (500 nM) or air (control) beginning 7 days after initiating ALI conditions and continuing for the remaining 21 days of differentiation. The cells were fixed and immunofluorescently stained for FoxJ1 and acetylated-tubulin to identify ciliated cells. Examination of the cells by confocal fluorescence microscopy (Fig 5A–5F) suggested that WCS treated cells without Gefitinib had fewer FoxJ1 positive, ciliated cells however WCS treated cells with Gefitinib were similar to air control cells. This was confirmed by quantitation of the the percent FoxJ1 positive cells (Fig 5G). These data suggested that WCS-mediated inhibition of ciliated cell differentiation is also mediated by EGFR signaling.

### Inhibition of EGFR signaling promotes recovery of FoxJ1 and ciliated cells expression after cessation of WCS exposure

**Fig 6:**
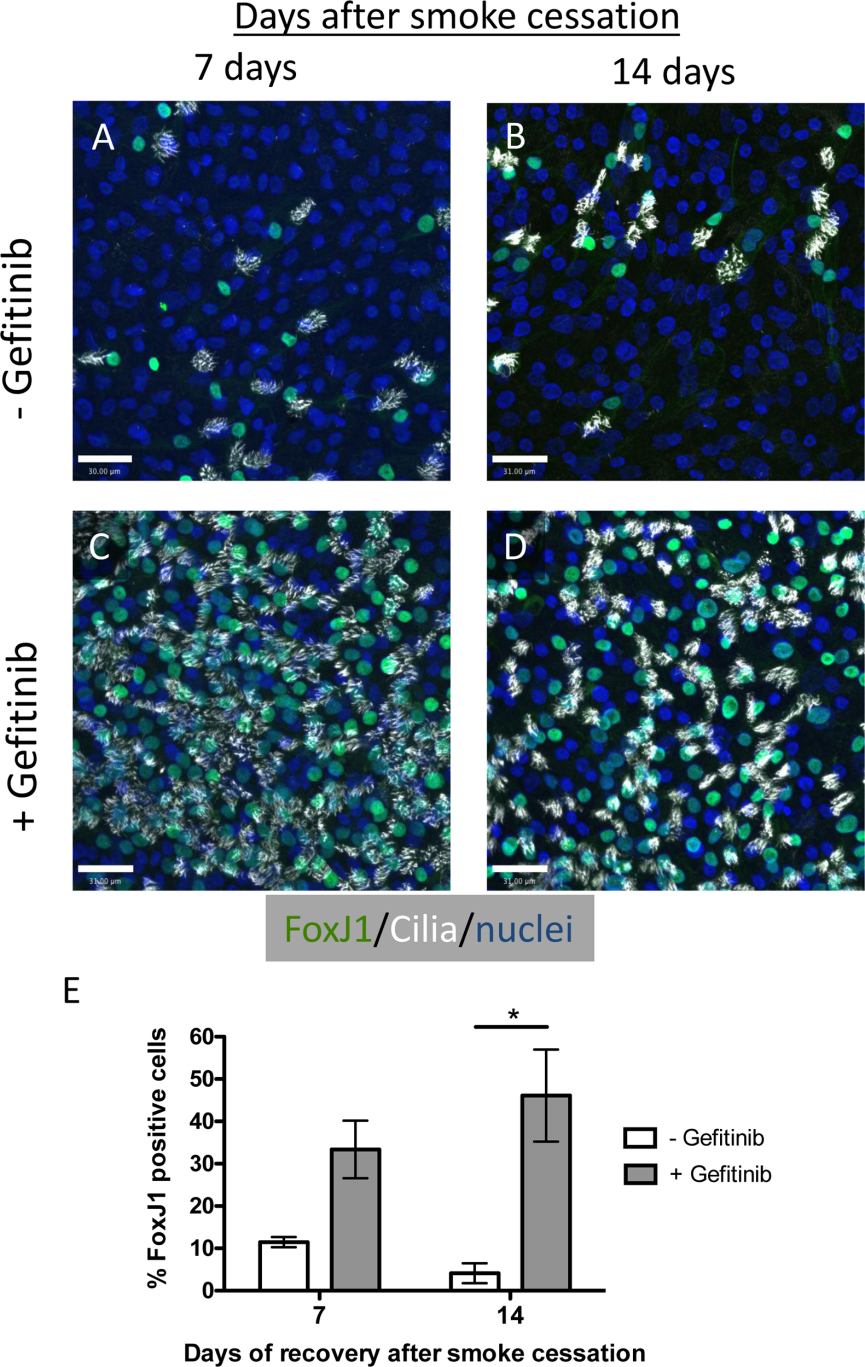
Inhibition of EGFR signaling promotes ciliated cell restoration after smoke exposure. **(A–D)** Representative confocal micrographs of WCS treated differentiated human airway epithelial cells treated without **(A, B)** or with **(C, D)** Gefitinib for 7 d **(A, C)**, or 14 d **(B, D)** after WCS treatment was stopped and stained for FoxJ1 (green), acetylated-tubulin (white) or nuclei (blue). Scale bar = 30 μm. **(E)** Quantification of the percent FoxJ1 positive cells cultured 7 and 14 d without (white bars) or with Gefitinib (grey bars) after WCS treatment was stopped. N = 2 for 7 d, N = 4 for 14 d. * P<0.05, Two tailed Student’s t test.

Since Gefitinib treatment prevented the loss of ciliated cells in WCS exposed differentiated NHBE cells and promoted ciliated cell differentiation in WCS exposed undifferentiated NHBE cells, we next examined whether Gefitinib could promote ciliated cell restoration in WCS treated differentiated cells after WCS exposure was discontinued. Differentiated NHBE cells were treated with WCS from 2 cigarettes every 2 d for 5 d for a total of 3 exposures as described above. After the third and final WCS exposure, the cells were allowed to recover for a period of 7 d or 14 d with or without Gefitinib (500 nM). The cultures were fixed and immunofluorescently stained for FoxJ1 and acetylated tubulin (Fig 6A–6D) and the percentage of FoxJ1 positive cells was quantified (Fig 6E). More ciliated cells were observed in the Gefitinib treated cells 7 d and 14 d after smoke cessation, there was a significant recovery of nuclear Foxj1 expressing cells in EGFR inhibitor treated cells compared to no treatment. These data suggest that WCS-induced EGFR signaling continued and prevents ciliated cell restoration after discontinuation of WCS exposure.

## Discussion

Long term cigarette smoke exposure has been shown to cause remodeling of the airway epithelium by reducing numbers of ciliated cells in animal models [23, 24] and in humans [25] however the mechanism of the loss of ciliated cells is not well studied and not completely understood. Most previous *in vitro* studies on the effects of cigarette smoke on ciliated cells were done using cigarette smoke extract. The experiments presented here examine the effects of WCS exposure on human ciliated epithelial cells grown in ALI cultures using the Vitrocell^®^ VC 10^®^ Smoking Robot. This method of smoke exposure was used because it more accurately reflects *in vivo* cigarette smoke exposure and can be tightly controlled in a reproducible manner. The results obtained from these experiments suggested that WCS causes reduction of ciliated cells in two ways. First, WCS caused a reduction in the number of existing ciliated cells in differentiated cultures during 7 days of exposure. The loss of ciliated cells was likely due to the de-differentiation of existing ciliated cells since the viability assays showed no significant cell death. In addition, the levels of MCIDAS and FoxJ1 mRNAs were reduced in cells exposed to WCS, consistent with the de-differentiation of ciliated cells. Similar results were reported for differentiated NHBE cells treated with CSE [24] indicating both methods of smoke exposure had similar effects on differentiated ciliated cells. These results are consistent with the model that WCS exposure represses MCIDAS causing the loss FoxJ1 expression, which in turn reduces the expression of genes necessary for maintaining cilia and ultimately cilia degradation. In addition, this model is also consistent with the observation of shorter cilia in the lungs of smokers [10, 26].

In addition to causing the acute loss of existing ciliated cells, we also showed that WCS inhibits differentiation of ciliated cells from basal stem cells. Similar observations were reported for CSE treatment [8, 12] and WCS treatment [27]. To investigate the mechanism WCS inhibition of ciliated cell differentiation, we measured effect on WCS on the expression of two genes that are necessary for ciliated cell differentiation, MCIDAS and FoxJ1. Previous studies showed that MCIDAS expression precedes and is required for FoxJ1 expression in the pathway of ciliogenesis, therefore WCS could be inhibiting the expression of either MCIDAS, FoxJ1 or both. In differentiated cells, we found a significant reduction in both MCIDAS and FoxJ1 mRNAs, however in differentiating cells we observed a reduction of FoxJ1 but not MCIDAS mRNA. This suggests WSC could be inhibiting the expression of MCIDAS to cause reduction in FoxJ1 expression that leads to de-differentiation of existing ciliated, but in differentiating cells WCS appears to be inhibiting FoxJ1 expression in an MCIDAS independent manner suggesting a possible different mechanism for controlling FoxJ1 transcription. Further studies are necessary to sort this out. These data suggest that long term remodeling and loss of ciliated cells in the airway due to WCS exposure results from the combination of acute ciliated cell loss of and inhibition of ciliated cell replacement by differentiation of basal cells.

More interestingly, we present data showing the EGFR inhibitor, Gefitinib, prevents both the smoke induced acute loss of ciliated cells and the inhibition of ciliated cell differentiation. Cigarette smoke induction of EGFR signaling is well established as a major factor in mucus overproduction [5, 6], disruption of epithelial cell junctions [19, 20] and gene expression [11], however its effects on ciliated cells has not been well established. The finding that Gefitinib prevents ciliated cell loss and promotes ciliated cell differentiation suggests that EGFR signaling also mediates WCS induced ciliated cell reduction. This also implies that EGFR inhibitors might be useful therapeutic agents to prevent the disruption of MCC due to ciliated cell loss and possibly to restore ciliated cells and MCC after cessation of CS exposure. This was tested by exposing differentiated NHBE cells to WCS and allowing the ciliated cells to recover with or without Gefitinib after WCS exposure was stopped. We found that replacement of ciliated cells after WCS exposure was terminated was much better in Gefitinib treated cells versus untreated cells. These data suggest that WCS-induced EGFR signaling persists after cessation of WCS exposure and inhibits basal cell differentiation to replace lost ciliated cells. Several mechanisms of smoke induced EGFR signaling have been reported including induction of EGFR ligand expression [28], proteolytic release of membrane tethered ligands [6, 29] and non-ligand mediated activation [21, 30] however, which if any of these is responsible for continued EGFR signaling after WCS cessation is yet to be determined. In conclusion, the data presented here indicate activation of EGFR signaling by WCS exposure promotes respiratory disease by causing ciliated cell loss and inhibiting ciliated cell replacement leading to reduced MCC and inefficient host defense. Furthermore, it suggests that inhibitors of EGFR tyrosine kinase activity and its downstream signaling molecules might be useful therapeutically to prevent airway ciliated cells loss and to promote ciliated cell replacement to treat and/or prevent airway diseases caused by WCS exposure.

## Supporting Information

S1 Fig Viability of differentiated NHBE cells during WCS treatment. Differentiated NHBE cells were treated with WCS from 2 cigarettes every 2 days beginning on Day 0. Viability was determined before WCS treatment, Day 0, and after 1 day (1 WCS treatment) and 7 days (3 WCS treatments) using the neutral red assay. Values are relative to air treated controls.
(TIF)

S2 Fig Viability of undifferentiated NHBE cells during WCS treatment. Undifferentiated NHBE cells were treated with WCS from 1 cigarette every 2 days beginning on Day 0. Viability was determined before WCS treatment, Day 0 (no smoke) and after 3 (2 WCS treatments), 7 (3 WCS treatments) and 14 days (6 treatments) using the neutral red assay. Values are relative to air treated controls.
(TIF)

S3 Fig Effect of Gefitinib on air-treated and WCS-treated differentiated NHBE cells. Differentiated NHBE cells were treated with air or WCS from 2 cigarettes every 2 days for 5 days (3 treatments). Two days after the 3rd treatment, the cells were washed with PBS, dissociated using trypsin and immediately fixed and permeabilized using Foxp3 /Transcription Factor Staining Buffer Set (eBioscience Cat.# 00-5523-00). Foxj1 was stained using goat anti Foxj1 (R&D Systems) and donkey anti-goat IgG (H+L) secondary antibody Alexa Fluor 647 conjugate (Life Technologies). After washing, the Foxj1 intracellular staining was analyzed using a LSR-II cytometer (BD) with FlowJo software (TreeStar). N = 5, * P<0.05, one way ANOVA with Tukey’s Multiple Comparison Test using Prizm 5 software.
(TIF)
