# Supplementary figures and images for: Gefitinib, an EGFR Tyrosine Kinase inhibitor, Prevents Smoke-Mediated Ciliated Airway Epithelial Cell Loss and Promotes Their Recovery
Source: PLoS One. 2016 Aug 17;11(8):e0160216. doi: 10.1371/journal.pone.0160216 (PMC4988636; doi:10.1371/journal.pone.0160216)

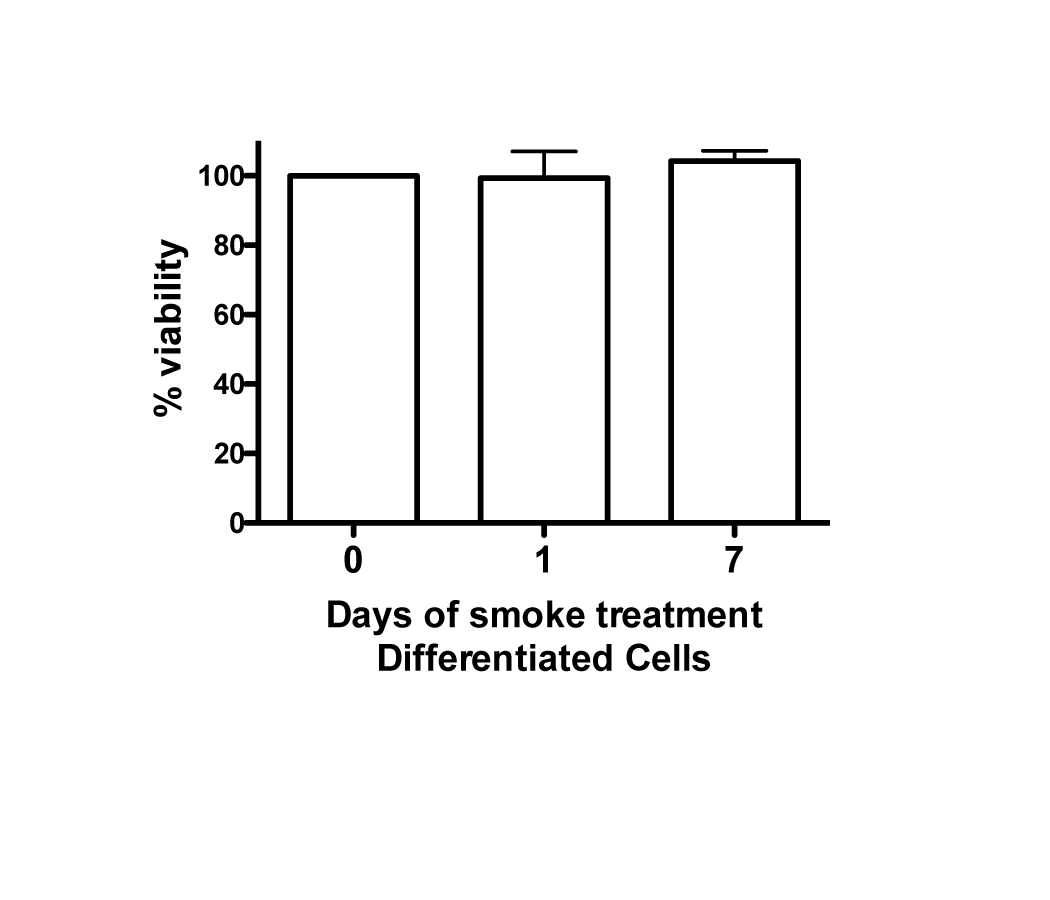

Supplement: S1 Fig — Differentiated NHBE cells were treated with WCS from 2 cigarettes every 2 days beginning on Day 0. Viability was determined before WCS treatment, Day 0, and after 1 day (1 WCS treatment) and 7 days (3 WCS treatments) using the neutral red assay. Values are relative to air treated controls. (TIF) [file pone.0160216.s001.tif]

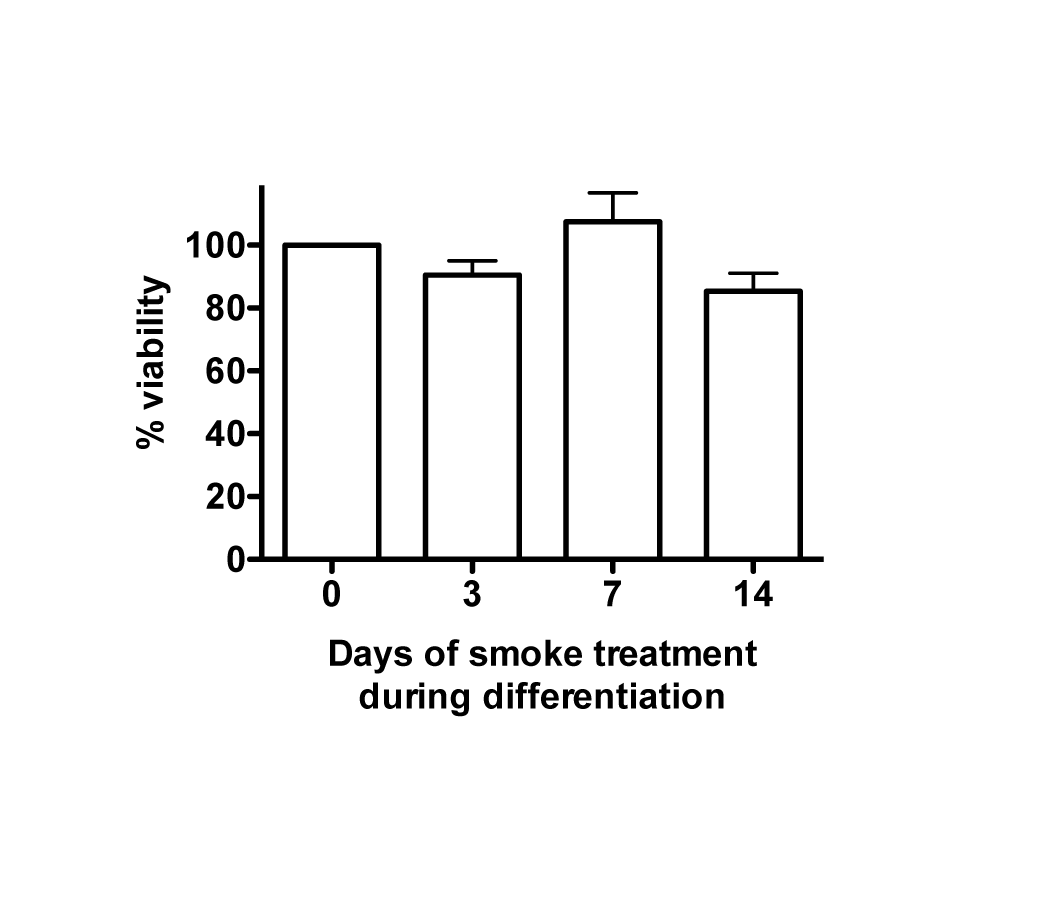

Supplement: S2 Fig — Undifferentiated NHBE cells were treated with WCS from 1 cigarette every 2 days beginning on Day 0. Viability was determined before WCS treatment, Day 0 (no smoke) and after 3 (2 WCS treatments), 7 (3 WCS treatments) and 14 days (6 treatments) using the neutral red assay. Values are relative to air treated controls. (TIF) [file pone.0160216.s002.tif]

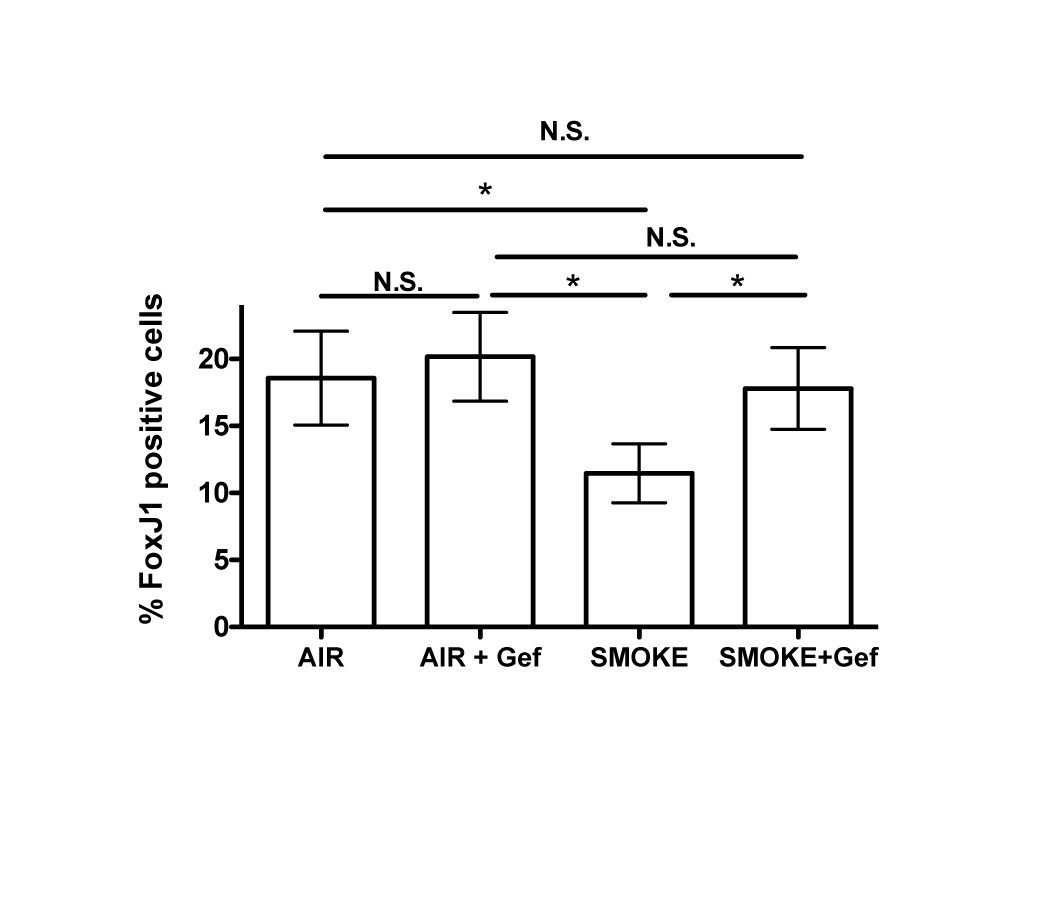

Supplement: S3 Fig — Differentiated NHBE cells were treated with air or WCS from 2 cigarettes every 2 days for 5 days (3 treatments). Two days after the 3rd treatment, the cells were washed with PBS, dissociated using trypsin and immediately fixed and permeabilized using Foxp3 /Transcription Factor Staining Buffer Set (eBioscience Cat.# 00-5523-00). Foxj1 was stained using goat anti Foxj1 (R&D Systems) and donkey anti-goat IgG (H+L) secondary antibody Alexa Fluor 647 conjugate (Life Technologies). After washing, the Foxj1 intracellular staining was analyzed using a LSR-II cytometer (BD) with FlowJo software (TreeStar). N = 5, * P<0.05, one way ANOVA with Tukey’s Multiple Comparison Test using Prizm 5 software. (TIF) [file pone.0160216.s003.tif]
